# Integrated analysis of DNA methylation and mRNA expression profiles to identify key genes in head and neck squamous cell carcinoma

**DOI:** 10.1042/BSR20193349

**Published:** 2020-01-24

**Authors:** Yu Jin, Xing Qin

**Affiliations:** 1 Department of Oral and Maxillofacial-Head and Neck Oncology, Ninth People’s Hospital, Shanghai Jiao Tong University School of Medicine, Shanghai 200011, P.R. China; 2 Department of General Dentistry, Ninth People’s Hospital, Shanghai Jiao Tong University School of Medicine, Shanghai 200011, P.R. China; 3 Shanghai Key Laboratory of Stomatology and Shanghai Research Institute of Stomatology, National Clinical Research Center of Stomatology, 200000, P.R. China

**Keywords:** biomarker, DNA methylation, GEO, head and neck squamous cell carcinoma, PPI, prognosis

## Abstract

DNA methylation has been demonstrated to play significant roles in the etiology and pathogenesis of head and neck squamous cell carcinoma (HNSCC). In the present study, methylation microarray dataset (GSE87053) and gene expression microarray dataset (GSE23558) were downloaded from GEO database and analyzed through R language. A total of 255 hypermethylated-downregulated genes and 114 hypomethylated-upregulated genes were finally identified. Functional enrichment analyses were performed and a comprehensive protein–protein interaction (PPI) network was constructed. Subsequently, the top ten hub genes selected by Cytoscape software were subjected to further analyses. It was illustrated that the expression level of CSF2, CTLA4, ETS1, PIK3CD, and CFTR was intimately associated with HNSCC. Survival analysis suggested that CTLA4 and FGFR2 could serve as effective independent prognostic biomarkers for HNSCC patients. Overall, our study lay a groundwork for further investigation into the underlying molecular mechanisms in HNSCC carcinogenesis, providing potential biomarkers and therapeutic targets for HNSCC.

## Introduction

Head and neck squamous cell carcinoma (HNSCC) is a prevalent malignancy with high morbidity and mortality, causing approximately 300,000 deaths each year all over the world [1]. It is widely acknowledged that alcohol and tobacco consumption are the two most important risk factors for HNSCC pathogenesis while human papillomavirus (HPV) infection has increasingly been recognized as an essential etiological factor [2]. Traditional clinical treatment methods for HNSCC included ablative surgery, chemotherapy, and radiotherapy while novel therapeutic measures such as anti-EGFR biotherapy have been developed in recent years. In spite of numerous modalities conducted on therapy methods, the life quality of HNSCC patients was not remarkably improved and the survival rate desperately remains at 50% [3]. This outcome may result from the high metastasis and recurrence rates of HNSCC and the delayed detection and diagnosis due to the asymptomatic nature of the early stage of this disease. Therefore, the development of novel and effective biomarkers is valuable to the early diagnosis and timely adequate treatments for HNSCC.

Epigenetic modifications, such as histone modification, noncoding RNA-related modifications and DNA methylation, have been determined as relevant factors in cancer progression among which DNA methylation especially drew the attention of more and more researchers [4]. The reason why it became the focus of a variety of studies may be its relative high frequency of occurrence and its ease of detection, making it a promising therapeutic target for different cancers. DNA methylation was demonstrated to be associated with many biological processes, including DNA damage repair, cell cycle period, apoptosis activities, and angiogenesis, making it a potent regulator in cancer progression [5]. Accumulating evidences have illustrated the involvement of aberrant gene methylation in tumor occurrence, progression, and metastasis [6]. Specifically, DNA methylation dysregulation includes hypermethylation with gene repression and hypomethylation resulting in the up-regulation of gene expression. Many cases have reported that gene silencing by the promotor hypermethylation of CpG islands is closely related with the inactivation of tumor suppressors, leading to the malignant transformation of various tumors. For example, methylation of TMEM176A was demonstrated to be related with tumor metastasis and served as an independent prognosis factor [7]. Another study revealed that DACT2 was repressed by promotor hypermethylation and pointed out the mediatory effect of DACT2 on papillary thyroid cancer proliferation and metastasis [8]. What is more, the aberrant promoter methylation of WT1, PAX5, PAX6, PYCARD, and GATA5 was observed in hepatocellular carcinoma and the correlation of them with clinical parameters suggested their potential applications in cancer diagnosis [9]. Despite the fact that rare studies concentrated on the DNA hypomethylation regulation in cancer progression, there still exist some findings about gene activation caused by promotor hypomethymation, such as the overexpression of Bcl-2, Cyclin D2, and R-ras [10–12].

Until now, several studies have explored the methylation profiles in HNSCC, manifesting possible biomarkers and therapeutic targets. A study conducted on primary tumors from HNSCC patients found a high proportion of p16 hypermethylation, presenting a promising marker for monitoring patients [13]. Furthermore, proliferation and colony formation assay illustrated the tumor suppressor role of SLC5A8. IRX1 and EBF3 in HNSCC were regulated by promoter hypermethylation [14]. What is more, CSPG4 was exploited as a robust biomarker for HNSCC and the up-regulation of CSPG4 by hypomethylation was intimately related with patient prognosis [15].

Although a good number of studies have reported potential methylation status-based markers for HNSCC, most of them were dependent on a limited sample size that may lead to biased and unreliable results. Also, a majority of the researches lack a comprehensive analysis of the profiles and enriched pathways of the interactive network. In the present study, we aimed to utilize multiple HNSCC datasets from Gene Expression Omnibus (GEO) and analyzed through integrated bioinformatics methods with the aim to figure out more precise and reliable screening results. Protein–protein interaction network (PPI) was constructed and hub genes were identified, GO and KEGG pathway analysis was further performed to shed light on the potential underlying mechanisms in HNSCC carcinogenesis.

## Materials and methods

### Microarray datasets

In the present study, we downloaded methylation microarray dataset (GSE87053) and gene expression microarray dataset (GSE23558) conducted on HNSCC samples from Gene Expression omnibus (GEO, https://www.ncbi.nlm.nih.gov/geo/). Specifically, there were a total of 11 HNSCC samples and 10 compared normal samples in GSE87053, while 28 HNSCC and 4 normal control specimens were collected in GSE23558. The methylation microarray dataset was based on GPL13534 platform (Illumina HumanMethylation450 BeadChip) while the gene expression dataset utilized GPL6480 platform (Agilent-014850 Whole Human Genome Microarray 4x44k G4112F).

### Data processing and differential expression analysis

The differential expression analysis of methylation status and gene expression level was performed through the minfi and limma package, respectively. Adjusted *P*-value less than 0.05 and β value more than 0.2 (hypermethylated genes) or less than −0.2 (hypomethylated genes) were defined as the criteria. For differentially expressed gene identification, adjusted *P*-value less than 0.01 and |logFC| more than 1 were set as the threshold. Subsequently, VennDiagram package in R was applied to identify overlapping hypermethylated-low expression genes and hypomethylated-high expression genes.

### GO and KEGG pathway enrichment analysis

The annotation, visualization, and integrated discovery (DAVID) database (https://david.ncifcrf.gov/) is a user-friendly online database on which a good number of genes could undergo integrated analysis including biological meaning and KEGG pathways. The gene oncology enrichment of candidate genes such as biological process, cellular component, molecular function, and KEGG enrichment pathway were analyzed by David and plotted by R language. The top 10 of the results were presented in the present study. The size of circle represents the gene counting number and the color represents the *P*-value of the enrichment analysis.

### Protein–protein interaction (PPI) network construction and module analysis

To have a more intuitive overview of the underlying associations between the candidate genes in HNSCC, an interactive PPI network was constructed in STRING database and visualized in Cytoscape. This functional network contributes to the understanding of the potential molecular mechanisms in HNSCC carcinogenesis. Then, the Molecular Complex Detection (MCODE) plugin in Cytoscape software was applied to screen out the most significant gene modules. Furthermore, the top 10 genes with the most connected degree was selected as hub genes by the cytoHubba plugin.

### Validation of hub genes expression level in GEPIA

To demonstrate the validity of bioinformatics analysis conducted on GEO datasets, we detected the relative mRNA level of hub genes in Gene Expression Profiling Interactive Analysis (GEPIA; http://gepia.cancer-pku.cn/), a web‐accessible tool based on the cancer genome atlas (TCGA) and GTEx data. In the present study, we subjected hub genes to GEPIA with a threshold of *P* < 0.05 and |logFC| > 1 to perform differential expression analysis.

### Relationship of hub genes expression with HNSCC patient prognosis

UCSC Xena (http://xenabrowser.net/) is a free online database from which users could obtain genomic data to investigate the potential correlations between specific gene expression and phenotypic variables such as TNM stage and patient prognosis. In the present study, we examined the relationship of hub genes expression with the survival of HNSCC patients based on the TCGA data in UCSC Xena. Patients were grouped into a relatively high expression group and a low expression group according to the median, and *P* < 0.05 was considered as statistically significant.

### Gene Set Enrichment Analysis (GSEA)

To further validate the involvement of CTLA4 or FGFR2 in KEGG pathways, we conducted GSEA analysis on the expression profiles of HNSCC samples in GSE23558. HNSCC samples were divided into relative high and low expression groups according to the relative level of CTLA4 or FGFR2, respectively. Subsequently, GSEA was performed in two groups to identify enriched pathways.

### Statistical analysis

All the statistical analyses were performed by the SPSS 23.0 software. Comparisons between two groups were conducted using Student’s two‐tailed *t* test. Kaplan–Meier survival analysis was utilized to explore the association of hub gene expression with HNSCC patient survival using log‐rank test. *P* < 0.05 was considered statistically significant in the present study.

## Results

### Identification of aberrantly methylated-differentially expressed genes in HNSCC

**Figure 1:**
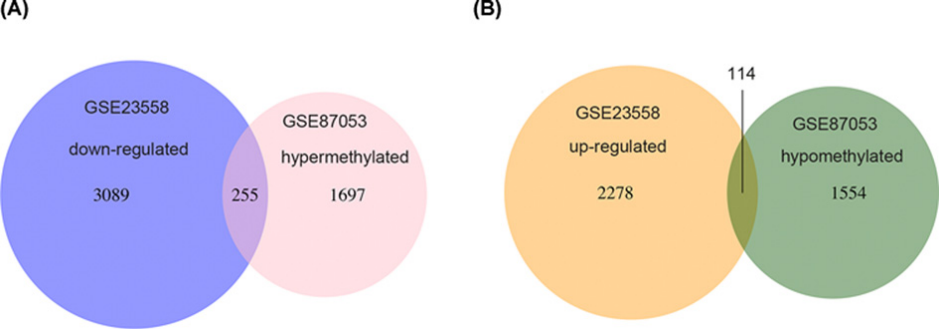
Identification of aberrantly methylated-differentially expressed genes in methylation microarray dataset (GSE87053) and gene expression dataset (GSE23558) (**A**) Venn diagram of both hypermethylated and down-regulated genes. (**B**) Venn diagram of both hypomethylated and up-regulated genes.

The aberrantly methylated-differentially expressed genes were identified by the methylation microarray dataset (GSE87053) and gene expression microarray dataset (GSE23558). As shown in Figure 1A,B, a total of 255 hypermethylated-downregulated genes and 114 hypomethylated-upregulated genes were identified.

### GO functional enrichment analysis

**Figure 2:**
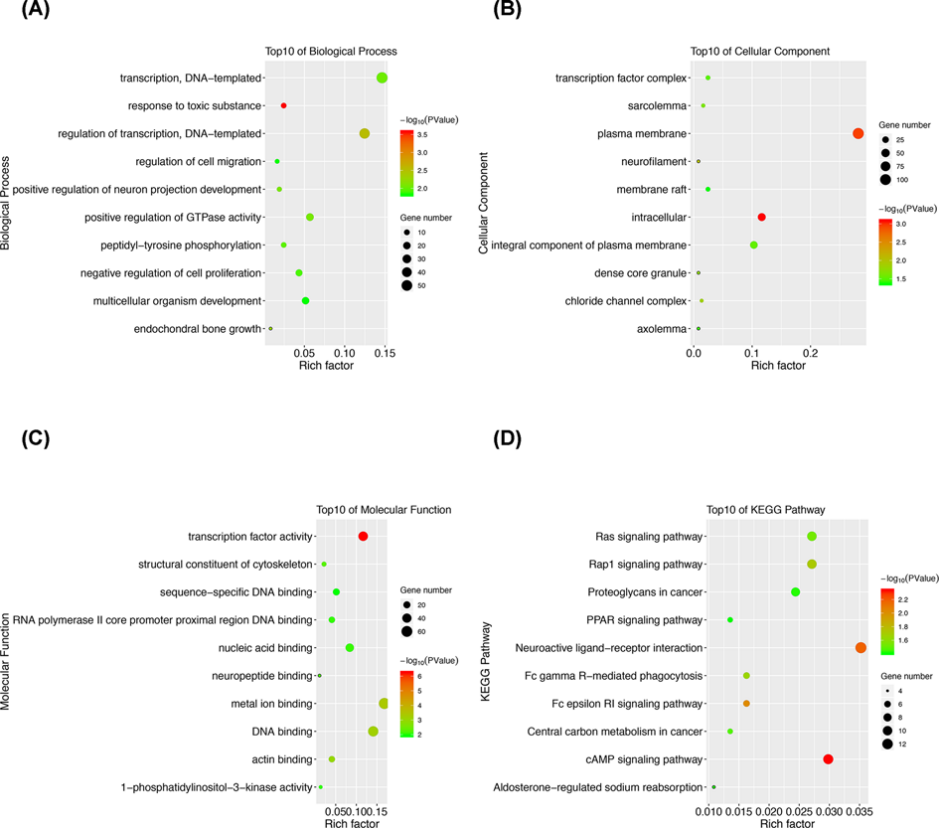
GO and KEGG pathway enrichment analysis of the aberrantly methylated-differentially expressed genes (**A**) GO analysis of the aberrantly methylated-differentially expressed genes in biological process. (**B**) GO analysis of the aberrantly methylated-differentially expressed genes in cellular component. (**C**) GO analysis of the aberrantly methylated-differentially expressed genes in molecular function. (**D**) KEGG pathway analysis of the aberrantly methylated-differentially expressed genes.

The top 10 results of gene enrichment in biological process, cellular component, and molecular function were obtained by David database and plotted in Figure 2. More specifically, the candidate genes were primarily enriched in transcription regulation, cell proliferation and migration regulation, and multicellular organism development (Figure 2A). While the terms enriched in the cellular component category included transcription factor complex, membrane, and intracellular region (Figure 2B). Furthermore, the molecular function category revealed enrichment in transcription factor activity, DNA binding, and actin binding (Figure 2C).

### KEGG pathway enrichment analysis

The top 10 results of KEGG pathway enrichment of the aberrantly methylated-differentially expressed genes were manifested in Figure 2D. As was shown, these genes were mainly enriched in Ras signaling, Rap 1 signaling, PPAR signaling, and cAMP signaling pathways.

### PPI network construction, module identification, and hub genes selection

**Figure 3:**
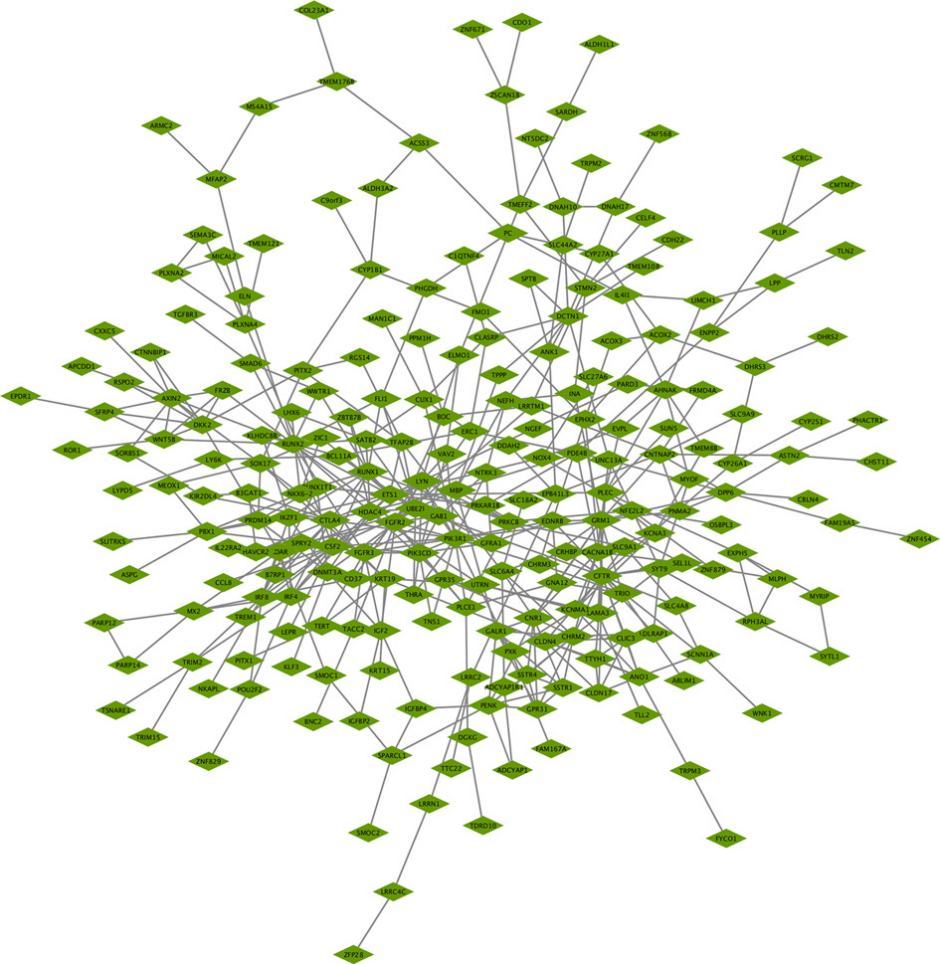
Protein–protein interactions (PPI) network of aberrantly methylated-differentially expressed genes Each node in the figure represents a protein and the edge between the nodes represents the interaction between the two proteins. The nodes with highest PPI scores were PIK3R1, CFTR, CSF2, GRM1, RUNX2, FGFR2, CTLA4, LYN, ETS1, and PIK3CD.

**Figure 4:**
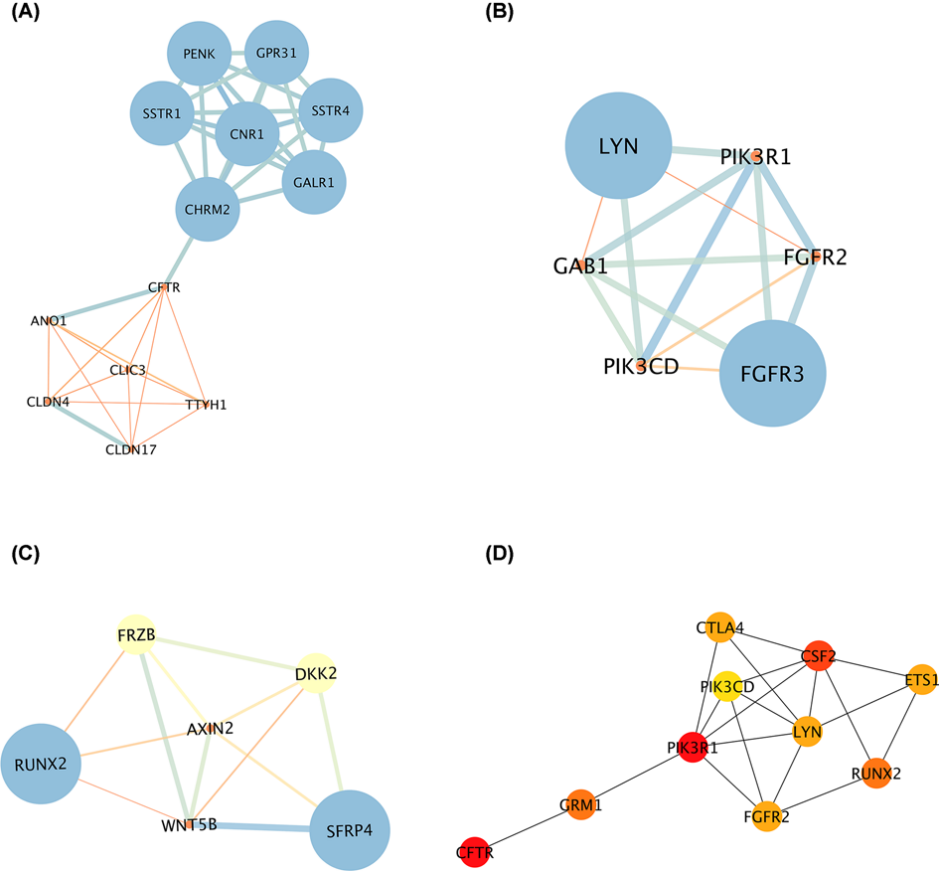
Gene module analysis and hub gene identification in the PPI network (**A–C**) The three most significant modules were screened out by the Molecular Complex Detection (MCODE) plugin in Cytoscape software. (**D**) The top 10 hub genes with the most connected degrees were identified by the CytoHubba plugin in Cytoscape.

A comprehensive and interactive PPI network of the aberrantly methylated-differentially expressed genes was established by the STRING database and visualized in Cytoscape software (Figure 3). Subsequently, the three most significant modules inside the PPI network were investigated by the MCODE application. More specifically, module 1 is composed of 13 nodes and 37 edges with the highest degree of 6.167 (Figure 4A). Module 2 contained 6 nodes and 14 edges while 6 nodes and 12 edges constituted module 3 (Figure 4B,C). Meanwhile, cytoHubba plugin was used to identify hub genes, the top 10 genes with the highest degree of connectivity were determined as hub genes in the present study. It was manifested in Figure 4D that PIK3R1 was the most significant gene with the greatest connectivity degree, followed by CFTR and CSF2.

### The transcription level of hub gene in GEPIA database

**Figure 5:**
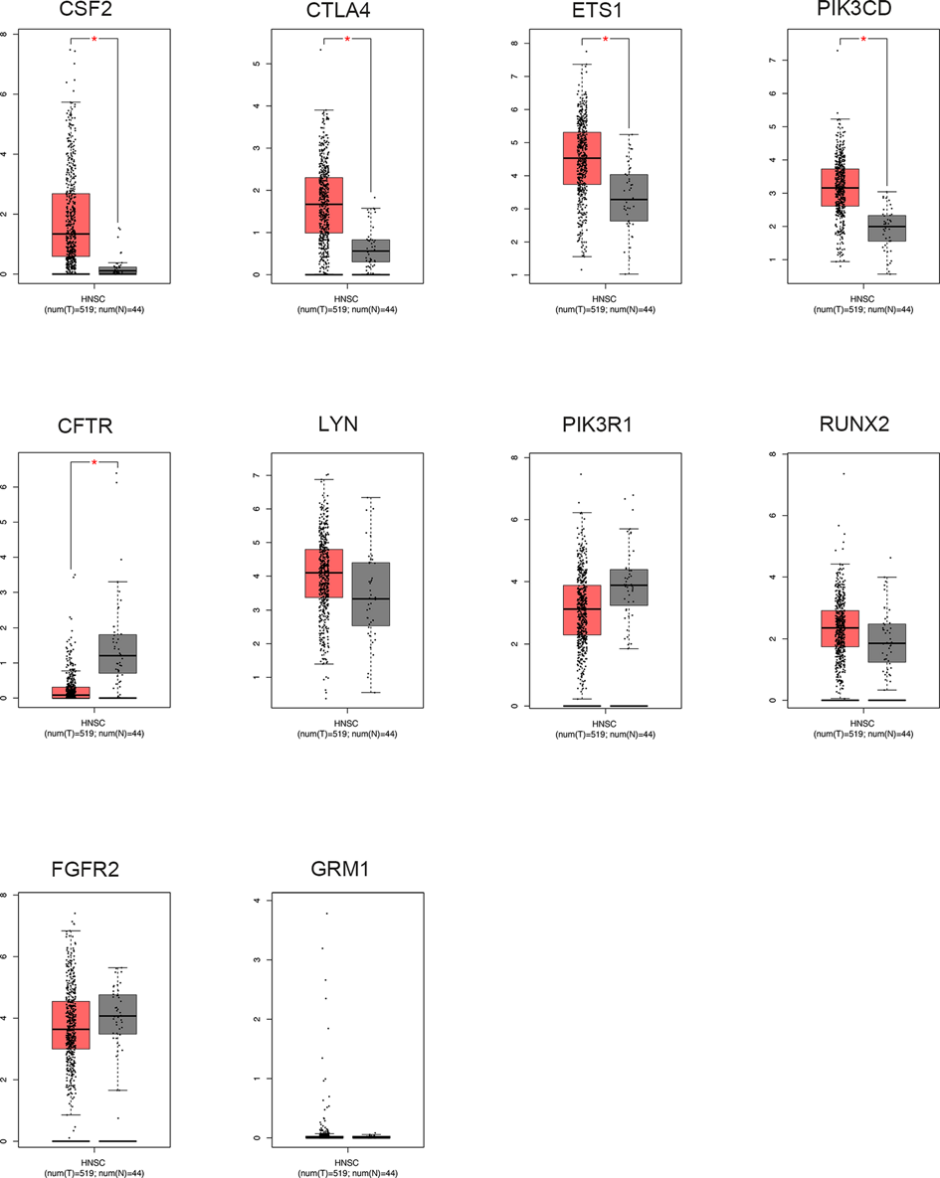
The transcription levels of the hub genes in HNSCC tissues and compared normal tissues were validated in GEPIA database The threshold was set as *P*-value = 0.05 and |log2(FC)| = 1. *, *P* < 0.05.

To validate the integrated bioinformatics analysis results, we detected the mRNA level of hub genes in GEPIA database. As was evidently shown in Figure 5, CSF2, CTLA4, ETS1, and PIK3CD from the hub genes were significantly up-regulated in HNSCC samples, implying the potential tumor suppressor roles of them. Meanwhile, an evident lower level of CFTR could be observed in HNSCC tissues when compared with normal controls, suggesting its oncogenic function.

### Survival analysis of hub gene expression in patients with HNSCC

Prognostic survival analysis was utilized to illuminate the influence of candidate gene expression on the prognosis of HNSCC patients. It could draw a conclusion from Supplementary Figure S1 that the expression level of CTLA4 and FGFR2 was closely correlated with the prognosis of HNSCC patients, while the remaining genes had no significant impact on patients’ overall survival. Moreover, the analysis of the relationship between hub gene with the relapse‐free survival of HNSCC patients showed that only FGFR2 notably affected the survival time of HNSCC patients (Supplementary Figure S2). To be details, patients with a relative higher level of FGFR2 had better relapse‐free survival compared with those with lower FGFR2 expression. The detailed information of the prognostic value of the hub genes was summarized in Table 1.

**Table 1:** The summary of prognostic value of ten hub genes and their related pathways

Gene name	The prognostic value for overall survival rate (*P* value)	The prognostic value for relapse-free survival rate (*P* value)	Top ten KEGG pathway enrichment
PIK3R1	0.787	0.582	cAMP signaling pathway Fc epsilon RI signaling pathway Rap1 signaling pathway Fc gamma R-mediated phagocytosis Ras signaling pathway Central carbon metabolism in cancer Proteoglycans in cancer Aldosterone-regulated sodium reabsorption
CFTR	0.826	0.365	cAMP signaling pathway
CSF2	0.649	0.417	Fc epsilon RI signaling pathway
GRM1	0.420	0.110	Neuroactive ligand–receptor interaction
RUNX2	0.299	0.545	
FGFR2	0.0141	0.0098	Rap1 signaling pathway Ras signaling pathway Central carbon metabolism in cancer
CTLA4	0.0004	0.231	
LYN	0.904	0.864	Fc epsilon RI signaling pathway Fc gamma R-mediated phagocytosis
ETS1	0.395	0.896	Ras signaling pathway
PIK3CD	0.622	0.595	cAMP signaling pathway Fc epsilon RI signaling pathway Rap1 signaling pathway Fc gamma R-mediated phagocytosis Ras signaling pathway Central carbon metabolism in cancer Proteoglycans in cancer Aldosterone-regulated sodium reabsorption

### Gene Set Enrichment Analysis (GSEA)

The enriched KEGG pathways of CTLA4 or FGFR2 in HNSCC were further analyzed by GSEA. As was shown in Supplementary Figure S3, some cancer-related gene set, such as “cell adhesion molecules cams”, “JAK-STAT signaling pathway”, “cytokine-cytokine receptor interaction” and several immune-related pathways including “antigen processing and presentation”, “natural killer cell mediated cytotoxicity”, and “T cell receptor signaling pathway” were finally screened out.

## Discussion

HNSCC is an intractable malignancy with survival rate staying at 50% in spite of enormous efforts and time focused on diagnosis and treatment improvement. It may be due to the fact that early stage HNSCC patients mostly present vague symptoms and unconspicuous physical changes. Therefore, investigating methods that could facilitate the early detection of HNSCC is of vital importance.

Epigenetic changes are reported to occur in almost all kinds of cancers, leading to the phenotype transformation without changing the DNA sequences. It was demonstrated that crucial genes involved in cancer initiation and progression could be regulated by epigenetic changes [16]. Until now, a variety of epigenetic modifications have been identified among which DNA methylation is acknowledged as a significant factor in carcinogenesis. Overall, it consists of two main parts, namely hypermethylation and hypomethylation. In many cases, the candidate tumor suppressor genes were regulated by promoter hypermethylation, resulting in a loss of function [17]. Accordingly, simultaneous silencing of multiple tumor suppressive genes may contribute to the malignant transformation of tumor formation. On the other hand, although not be completely studied, abnormal gene activation by hypomethylation may also play critical roles in the development and progression of tumors. More importantly, when compared with other popular molecular biomarkers such as mRNA and protein alterations, DNA methylation changes occur in an earlier stage and with a more stable status without being influenced by transient signals [18]. Therefore, exploring the DNA methylation profiling is a rational and sensible choice for discovering robust and reliable biomarkers for cancer.

A good number of studies conducted on various tumor types have illustrated the significance of DNA methylation in gene regulation. For example, DNA methylation of SHOX2 was exploited as an effective biomarker to distinguish between malignant lung disease and controls with a relative high sensitivity [19]. Furthermore, multiple genes were validated to be mediated by aberrant DNA methylation, acting as diagnostic, treatment, and prognostic markers for esophageal squamous carcinoma [16]. Also, the methylation status of ZNF671 was utilized as an independent marker for predicting ovarian cancer recurrence [20]. As with these different kinds of tumors, a series of methylation-related gene dysregulation has been detected in HNSCC, including p16, CDH1, DAPK and MGMT [21], implying their potential functions in HNSCC pathogenesis.

The emerging microarray and next-generation sequencing technology provided researchers a more comprehensive and in-depth overview of human genome sequences. A majority of the findings were dependent on either new microarray conducted on tissue samples or integrated analysis based on existing microarray data. Recently, increasingly combined analysis of DNA methylation status and transcriptional expression in various tumors has been performed. A study made a bioinformatics-based discovery of possible aberrantly methylated-differentially expressed genes and pathways in colorectal cancer so as to unravel the carcinogenesis process [22]. Another study conducted integrated analysis on TCGA data to get a better understanding of molecular mechanisms involved in esophageal squamous cell carcinoma, contributing to a more precise prognosis detection [23]. What is more, a study concentrated on lung adenocarcinoma found out several hub genes that could be employed to facilitate early diagnosis and accurate prognosis assessments of patients [24]. In the present study, methylation microarray dataset (GSE87053) and gene expression microarray dataset (GSE23558) were analyzed through R language and a total of 255 hypermethylated-down-regulated genes and 114 hypomethylated-upregulated genes were identified. Functional enrichment analyses including go oncology and KEGG pathway analysis were performed. It was concluded that the candidate genes were primarily enriched in transcription regulation, cell proliferation, and migration regulation; all were universally proved to be closely correlated with tumor progression. Moreover, results showed that a majority of genes showed enrichment in Ras signaling, Rap 1 signaling, PPAR signaling, and cAMP signaling pathways. The Ras proteins were among the first proteins demonstrated to possess the ability of mediating cell growth. Ras gene mutations are presented in nearly 30% of all human cancers and result in constitutive signaling activation, thereby promoting cell proliferation and inhibiting cell apoptosis [25]. Increasingly studies focused on inhibiting Ras-mediated signaling activation with the purpose to invent innovative therapeutic approaches [26]. Ras-associated protein-1 (Rap1), a member of the Ras-related protein family, participates in the regulation of several basic cellular functions including cell adhesion, cellular migration and invasion in different cancers [27]. It was reported that Rap1 depletion in non-small cell lung cancer led to reduced cancer cell growth and increased cisplatin sensitivity, suggesting the possibility of utilizing Rap1 as effective marker and therapeutic target [28]. The cyclic AMP (cAMP) signaling pathway is activated by cAMP, whose elevated levels could activate a variety of target molecules, thereby mediating different kinds of biological processes, such as cell metabolism, cell proliferation, and metastasis [29]. A study also suggested that modulating cAMP signaling could remarkably improve the chemotherapy and radiotherapy efficacy, contributing to the development of novel therapeutic strategies to enhance the cancer patient prognosis [30]. Peroxisome-proliferator-activated receptors (PPARs), a group of multifunctional transcription factors, mainly composed of three proteins, PPARα (NR1C1), PPARβ/ δ (NR1C2), and PPARγ (NR1C3). Researchers found that PPARs intimately involve in the epithelial–mesenchymal transition process of tumor formation, an essential process regulating cancer migration and metastasis [31]. Other literature also illustrated its modulatory functions in complex pathways underlying tumorigenesis. Clinically, application of PPARs agonists has been proved to decrease the incidence of tumor initiation [32]. In summary, all the enriched pathways screened out by bioinformatics tools were previously validated to be associated with cancer progression, laying a solid foundation for further investigation.

Subsequently, ten hub genes selected by Cytoscape software were subjected to further analyses. It was illustrated that there were significant differences between HNSCC and compared normal samples regarding the expression of CSF2, CTLA4, ETS1, PIK3CD, and CFTR. Colony Stimulating Factor 2 (CSF2), a granulocyte macrophage-colony stimulating factor, was illustrated to have intimate associations with diverse cancers. Overexpression of CSF2 was demonstrated to be implicated with advanced tumor status and poor prognosis in urothelial carcinoma and involved in several cancer-related pathways [33]. In skin cancers, CSF2 could regulate VEGFR secretion and recruit tumor-associated macrophages to perform its anti-proliferative ability [34]. Cytotoxic T lymphocyte antigen 4 (CTLA4), a negative regulator of T cells leading to its inactivation, is a cancer-related immune checkpoint [35]. Some studies suggested a combination of anti-CTLA4 therapy with anti-PDL1 therapy, a newly developed immunotherapy with high efficacy, implying the importance of CTLA4 in cancer progression [36]. ETS1, an essential member of the ETS transcription factors, participated in a variety of crucial biological processes underlying tumor progression, such as cell invasion, epithelial–mesenchymal transition, angiogenesis, and drug resistance [37]. Furthermore, the cross-talk of ETS1 with microenvironment makes it an essential factor for tumor metastasis, serving as a candidate therapeutic target [38]. PIK3CD, whose ectopic expression could affect the biological phenotypes of multiple cancers, was reported to regulate the PI3K signaling pathway [39]. Also, further analysis suggested it could be employed as an independent predictor for the prognosis of cancer patients [40]. Cystic fibrosis transmembrane conductance regulator (CFTR), a gene mainly expressed in epithelial cells, was found to play pivotal roles in both physiological processes and pathological stages [41]. Specifically, it was illustrated to mediate the innate immune process, which presents more and more impact on cancer regulation due to the emergence of immunotherapy [42]. In particular, the silencing of CFTR by promoter hypermethylation in a majority of tumor types enhanced the reliability of our bioinformatics-based analysis [43]. In our study, survival analysis displayed great differences in the survival condition of HNSCC patients between the relative high and low level of CTLA4 and FGFR2. Cytotoxic T lymphocyte antigen 4 (CTLA4), the first immune-checkpoint receptor to be clinically targeted, is expressed exclusively on T cells and primarily regulates T-cell activation. On account of the increasing importance of immune system in tumorigenesis, CTLA4 may play an indispensable role in cancer therapy. A study showed that drugs blocking and deactivating CTLA4 could help T cells find and attack cancer cells and proved to be effective in cancer treatment [44]. Anti-CTLA4 therapy could strongly enhance the amplitude of vaccine-induced antitumor responses in many poorly immunogenic tumor models [45]. Furthermore, some studies suggested a combination of anti-CTLA4 therapy with anti-PDL1 therapy, which has been demonstrated to be of high efficacy [36]. Fibroblast growth factor receptor 2 (FGFR2), a kind of tyrosine kinases, presented frequent SNPs and point mutations in breast cancer, and these epigenetic changes increased the risk of tumor formation. The genetic alterations of FGFR2 were reported to enhance downstream signaling and are associated with cancer development and progression [46,47]. For example, overexpression of FGFR2 could promote the proliferation and survival of gastric cancer cells through activating MAPK/ERK and PI3K/AKT signaling pathways [48]. Another study illustrated that FGFR2 could enhance the phosphorylation activity of RSK2 and regulate the migratory ability of human mammary epithelial cells [49]. Moreover, great attention has been paid on inventing FGFR2-targeted specific inhibitors for developing more precise and effective therapies for cancer [50,51]. Overall, CTLA4 and FGFR2 could be exploited to make innovative strategies for preventing or treating HNSCC.

Finally, GSEA results screened out several pathways involved in cancer progression, laying a solid foundation for the possible functional roles of CTLA4 or FGFR2 in HNSCC, encouraging more in-depth investigation in the future. Overall, our findings may remarkably contribute to the understanding of molecular mechanisms underlying HNSCC so as to facilitate therapeutic decision making, risk stratification, and prognosis prediction for HNSCC patients.

In fact, epigenetic therapy has been widely applied in multiple kinds of cancers among which DNA methylation inhibitors have promising prospect [52]. Specifically, 5-aza-2′-deoxycytidine (5-Aza-CdR), a kind of DNA methylation inhibitor, was approved for the clinical treatment of myelodysplastic syndrome [53]. Reactivation of candidate tumor suppressor genes by DNA methylation inhibitors also opens a new frontier for the treatment of cholangiocarcinoma [54]. Thus, illuminating the DNA methylation profiling in HNSCC and inventing appropriate therapy based on DNA methylation modulation may be of great value.

However, there exist some limitations in the present study. First, validation of the selected aberrantly methylated differentially expressed genes was only implemented on TCGA data. Furthermore, *in vitro* experiments remain to be conducted to confirm the findings of the identified genes and to further investigate potential molecular mechanisms.

In conclusion, with the methylation microarray and gene expression microarray, the present study provides a bioinformatics-based discovery of abnormal DNA methylated differentially expressed genes, which may have profound impact on HNSCC development and progression. Hub genes including CSF2, CTLA4, ETS1, PIK3CD, and CFTR may act as efficient biomarkers for diagnosis while CTLA4 and FGFR2 serve as promising biomarkers for the prognosis evaluation of HNSCC.

## Supplementary Material

Supplementary Figures S1-S3

## Abbreviations

5-Aza-CdR: 5-aza-2′-deoxycytidine
CFTR: cystic fibrosis transmembrane conductance regulator
FGFR2: fibroblast growth factor receptor 2
HNSCC: head and neck squamous cell carcinoma
PPI: protein–protein interaction
