# Supplementary material for: Integrated analysis of DNA methylation and mRNA expression profiles to identify key genes in head and neck squamous cell carcinoma
Source: Biosci Rep. 2020 Jan 24;40(1):BSR20193349. doi: 10.1042/BSR20193349 (PMC6981101; doi:10.1042/BSR20193349)
Supplement: Supplementary Figures S1-S3 [file BSR-2019-3349_supp.pdf]

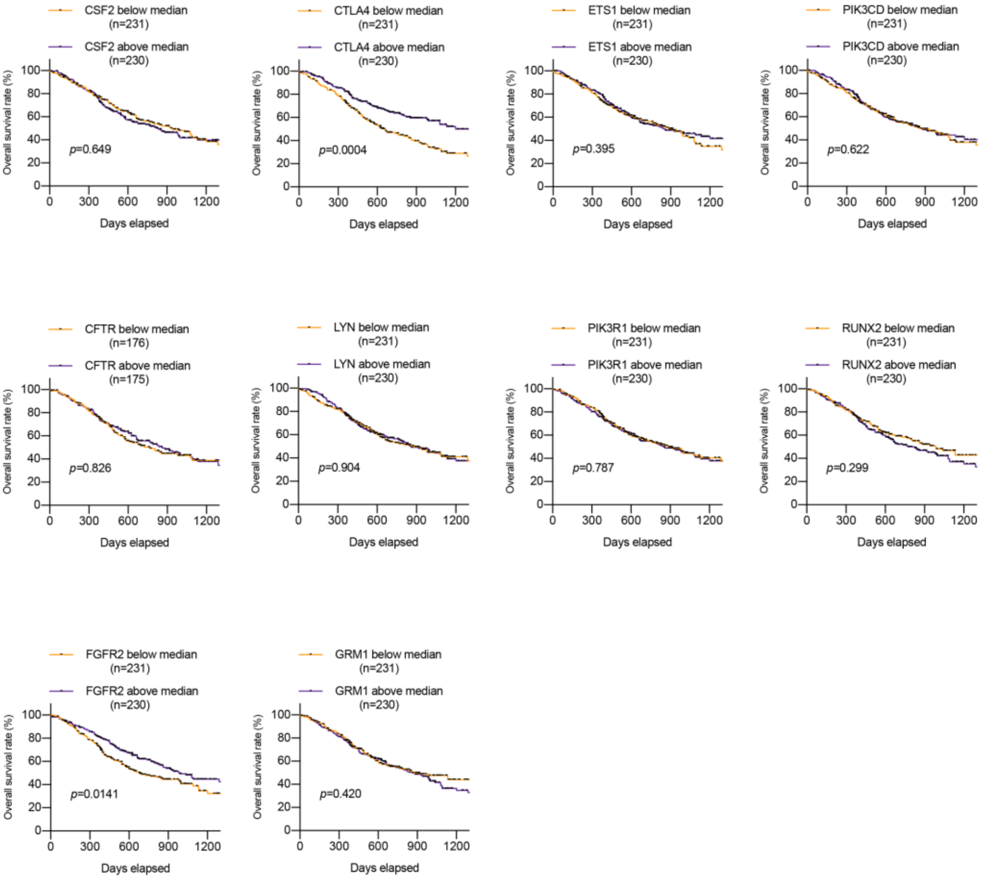

Figure S1. The prognostic value of hub genes in the overall survival of HNSCC patients based on TCGA data.

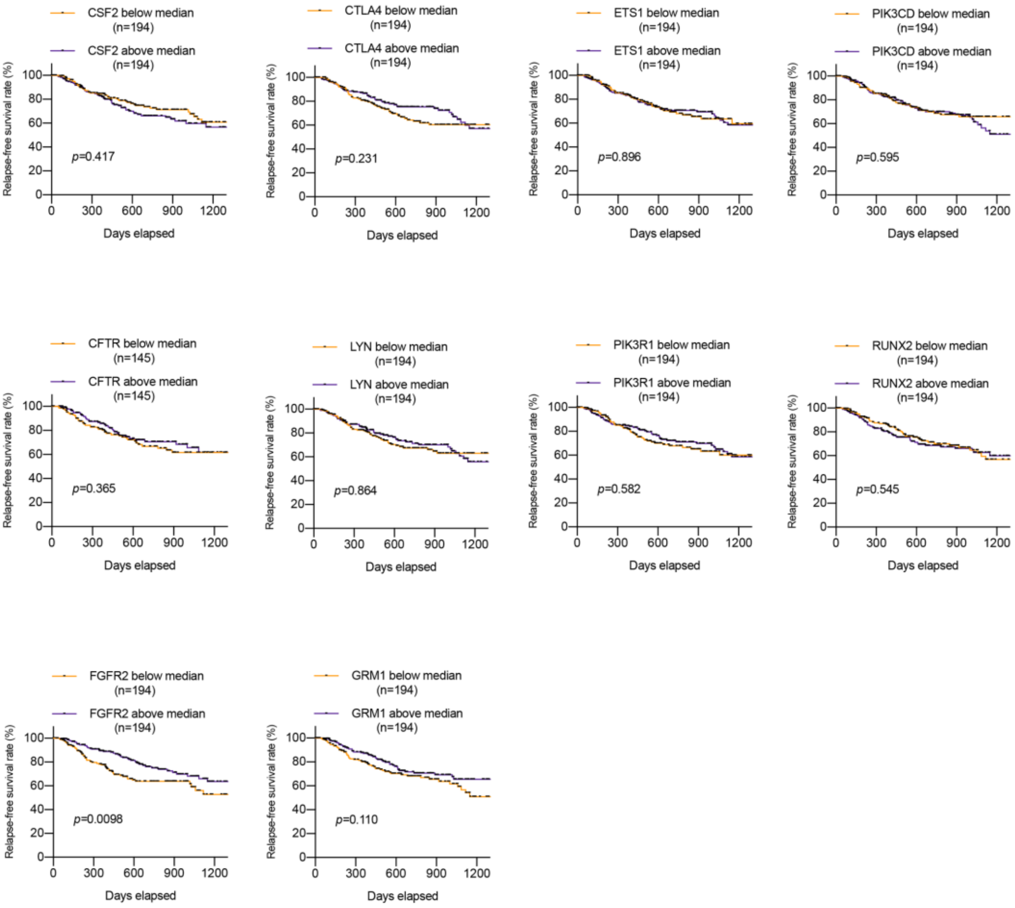

Figure S2. The relationship of hub genes with the relapse-free survival of HNSCC patients based on TCGA data.

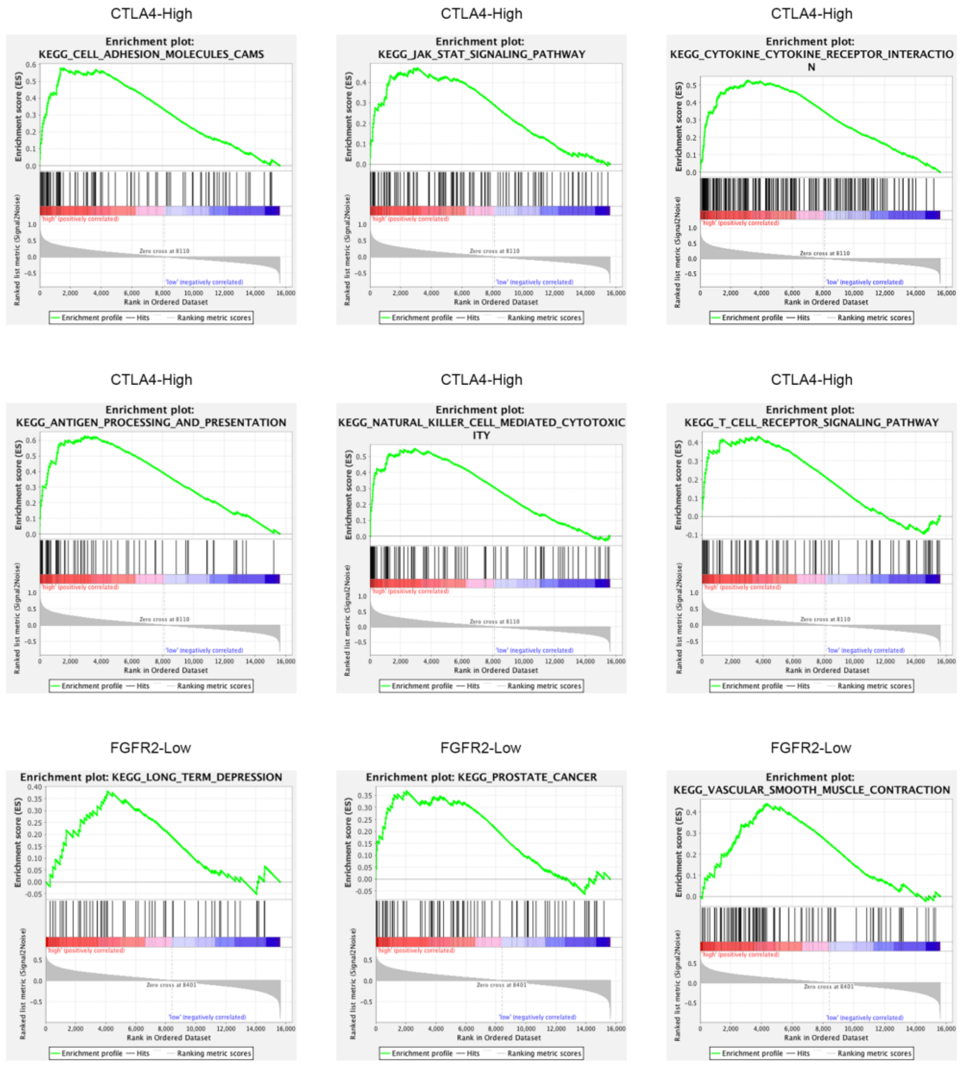

Figure S3. Gene set enrichment analysis (GSEA) for expression profiles in HNSCC samples with relative higher expression of CTLA4 or FGFR2.
